# Effect of stem structural characteristics and cell wall components related to stem lodging resistance in a newly identified mutant of hexaploid wheat (*Triticum aestivum* L.)

**DOI:** 10.3389/fpls.2022.1067063

**Published:** 2022-11-22

**Authors:** Darshana Bisht, Naveen Kumar, Yogita Singh, Rashmi Malik, Ivica Djalovic, Narendra Singh Dhaka, Neeraj Pal, Priyanka Balyan, Reyazul Rouf Mir, Vinay Kumar Singh, Om Parkash Dhankher, Upendra Kumar, Sundip Kumar

**Affiliations:** ^1^ Molecular Cytogenetics Laboratory, Department of Molecular Biology & Genetic Engineering, College of Basic Science & Humanities, Govind Ballabh Pant University of Agriculture and Technology, Pantnagar, India; ^2^ Department of Molecular Biology & Biotechnology, College of Biotechnology, Chaudhary Charan Singh Haryana Agricultural University, Hisar, India; ^3^ Department of Genetics & Plant Breeding, College of Agriculture, Govind Ballabh Pant University of Agriculture & Technology, Pantnagar, India; ^4^ Institute of Field and Vegetable Crops, National Institute of the Republic of Serbia, Novi Sad, Serbia; ^5^ Department of Botany, Deva Nagri Post Graduate College, CCS University, Meerut, India; ^6^ Division of Genetics and Plant Breeding, Sher-e-Kashmir University of Agricultural Sciences and Technology of Kashmir (SKUAST-Kashmir), Srinagar, India; ^7^ Department of Mechanical Engineering, College of Engineering, Govind Ballabh Pant University of Agriculture and Technology, Pantnagar, India; ^8^ Stockbridge School of Agriculture, University of Massachusetts, Amherst, MA, United States

**Keywords:** wheat, mutagenesis, stem strength, lodging, FTIR, lignin, UTM

## Abstract

In wheat, lodging is affected by anatomical and chemical characteristics of the stem cell wall. Plant characteristics determining the stem strength were measured in lodging tolerant mutant (PMW-2016-1) developed through mutation breeding utilizing hexaploid wheat cultivar, DPW-621-50. Various anatomical features, chemical composition, and mechanical strength of the culms of newly developed lodging-tolerant mutant (PMW-2016-1) and parent (DPW-621-50), were examined by light microscopy, the Klason method, prostate tester coupled with a Universal Tensile Machine, and Fourier Transform Infrared Spectroscopy. Significant changes in the anatomical features, including the outer radius of the stem, stem wall thickness, and the proportions of various tissues, and vascular bundles were noticed. Chemical analysis revealed that the lignin level in the PMW-2016-1 mutant was higher and exhibited superiority in stem strength compared to the DPW-621-50 parent line. The force (N) required to break the internodes of mutant PMW-2016-1 was higher than that of DPW-621-50. The results suggested that the outer stem radius, stem wall thickness, the proportion of sclerenchyma tissues, the number of large vascular bundles, and lignin content are important factors that affect the mechanical strength of wheat stems, which can be the key parameters for the selection of varieties having higher lodging tolerance. Preliminary studies on the newly identified mutant PMW-2016-1 suggested that this mutant may possess higher lodging tolerance because it has a higher stem strength than DPW-621-50 and can be used as a donor parent for the development of lodging-tolerant wheat varieties.

## Introduction

The term “lodging” refers to the persistent displacement of shoots from their vertical or upright position due to certain specific internal and environmental factors. It is more common in cereal crops, particularly wheat and rice, and gives rise to significant losses related to reduced quality, yield, and harvest efficiency (Fischer and Stapper, 1987; Berry et al., 2003). Cereal crops generally exhibit stem and root lodging (Berry et al., 2004). Stem lodging is due to the bending and breaking of the lower culm internodes consisting of thin diameter and less tensile strength (Islam et al., 2007), whereas root lodging (anchorage failure) results from poor root-soil integrity, causing the straight stalks to bend or tumble from the tip (Baker et al., 1998). Lodging in cereal crops tends to be higher when the crop is near harvest (Shah et al., 2017). For example, in winter wheat, the crop may lodge at any time between the emergence of the ear and the grain maturity. In wheat, approximately 31% and 25% reduction in yield was recorded after imposing the lodging at the time of ear emergence and the milking stage respectively. In the most serious case, lodging resulted in up to 80% yield loss (Berry et al., 2007; Acreche and Slafer, 2011; Muhammad et al., 2020).

Lodging is a complex process that is affected by environmental agents (wind, hail, rain, topography, and soil type), morphological characteristics, chemical composition, and the anatomical features of culms. Morphological characteristics such as stem length, length and diameter of basal internodes, the diameter of culms, number of tillers, and plant height, are closely associated with the nature and extent of lodging (Kelbert et al., 2004; Kong et al., 2013; Okuno et al., 2014; Berry and Berry, 2015; Yadav et al., 2017). Anatomical features such as the thickness of the culm wall, the number of vascular bundles, the diameter of vascular bundles and the culm cavity, and thickness from the epidermis to the sclerenchyma layer are associated with lodging resistance (Xu et al., 2000; Kong et al., 2013). When it comes to chemical composition, lignin has long been thought to be the principal cause of differences among varieties in bending and lodging resistance. Many studies have observed a connection between lignin content and stem-breaking strength in different cultivars of wheat (Li et al., 2021; Dong et al., 2022). It has also been reported that increased hemicellulose and lignin concentration has a synergistic effect on stem-breaking resistance (Kong et al., 2013; Okuno et al., 2014; Zheng et al., 2017). The lodging effects have been studied in various crops, using different techniques and the most common is the one that includes the pushing of the resistance from the lower parts of the plant (Terashima et al., 1992; Berry et al., 2003).

Although the composition and characteristics of plant cell walls have been postulated to influence the mechanical strength of rice, no such effects on lodging resistance were studied in wheat. Moreover, the role of these cell wall components in stem strength and the processes governing their interaction is yet to be thoroughly understood. Despite having identical quantities of wall polymers, other factors i.e., hemicellulose monosaccharides, the crystallinity of cellulose, and lignin monomer composition may have a significant impact on lignocellulosic biomass (Park et al., 2010; Wu et al., 2013). However, only a few studies have shown the effects of the structural composition of wall materials on stem strength.

This study was conducted to examine the morphological features, cell wall composition, lignocelluloses, and anatomical characteristics of the newly developed lodging tolerant mutant wheat influencing resistance to stem breaking using light microscopy, Klason method, prostate tester coupled with Universal Tensile Machine (UTM), and Fourier Transform Infrared Spectroscopy (FTIR). Ultimately, our main aim was to develop a lodging-resistant variety for reliable agricultural production.

## Material and methods

### Plant materials

The lodging tolerant mutant (PMW-2016-1) was developed from hexaploid wheat (*Triticum aestivum* L.) cultivar DPW-621-50 exposed to EMS (Ethyl methane sulfonate). The seeds of DPW-621-50 were treated with 0.7% EMS (Ethyl methane sulfonate). The mutant, PMW-2016-1, was identified through the manual screening of the M_4_ population and maintained up to the M_6_ generation. The manual identification was based on the morphological features like more sturdiness and culm diameter of the mutant (PMW-2016-1) with respect to the parent (DPW-621-50) and then other physical attributes related to lodging tolerance were measured here in this study. The M_6_ population along with the parent (DPW-621-50) was planted in 2018-2019 in 2.0-meter rows with a 30 cm row-to-row and a 10 cm plant-to-plant distance. A standard package of agronomic practices was followed. At maturity of both (PMW-2016-1) and (DPW-621-50), second internodes from the basal stems were collected before harvesting and fixed in a fixative solution of formyl aceto-alcohol (FAA) (50% ethanol, 10% formalin, 5% glacial acetic acid, and 35% double distilled water). Samples were incubated in this fixative solution for 48 hr and then transferred to 70% ethanol for long-term storage. The stem samples collected were also used to measure other attributes.

### Analysis of morphological and anatomical features

From the middle of each internode, transverse sections of 20 µm were freshly cut and then viewed under a light microscope, fitted with a digital camera (Zeiss AxioCam MRm 1.4 CCD). The thickness of the stem wall and the width of the outer radius of the stem were measured under the microscope. Under a light microscope, the number of vascular bundles was counted. The number of large vascular bundles in parenchyma tissues and small vascular bundles distributed under the epidermis was analysed.

### Histochemical staining

A Weisner reaction was carried out to histochemically locate the lignin according to the standard protocol (Mitra and Loque, 2014). The fixed samples were dehydrated in a graded ethanol series and finally in a xylene series. Fresh hand-cut sections (~20 µm) of PMW-2016-1 and DPW-621-50 culms were incubated in phloroglucinol solution (2 volume 3% in ethanol:1 volume concentrated HCl) for 2 min. For toluidine blue staining, 0.02% aqueous solution of toluidine blue O was used to stain the free-hand sections for 2 min (Li et al., 2003). The sections were rinsed with distilled water 3-4 times until the solution was clear. These sections were then mounted with DPX and observed under a bright-field microscope. Sections were viewed under magnification of 10X and 40X for vivid visualization of lignin distribution across the stem and to differentiate the major tissues. Digital images were scored/taken using an Axiocam MRm camera.

### Estimation of lignin content

The second internodes of PMW-2016-1 and DPW-621-50 plants were harvested at maturity. The Klason lignin content was estimated by incubating the crushed internodes with 72% sulfuric acid for 1 hour and extracted two times with 72% sulfuric acid in water at 65°C for 30 min followed by rinsing with water and overnight drying of the residue at 80°C. After drying, the lignin content was measured following the method described by Kirk and Obst (1988).

### Mechanical strength of lodging-tolerant mutants

A prostate tester was used to measure the mechanical strength of stems of PMW-2016-1 and DPW-621-50. The stem strength/lodging resistance was measured according to the method described by Xiao et al. (2002). The force exerted to break the culm internodes (bending stress) of PMW-2016-1 and DPW-621-50 was investigated by using a universal testing machine (AMT-SC-01521). To reduce the error in sampling, internodes of equal length and width were used for the measurements. To avoid any inaccuracies, three replications were taken for each measurement and the values were averaged.

### Fourier Transform Infrared Spectroscopy (FTIR) Analysis

The internodes of two genotypes of wheat stems were ground into fine powder in liquid nitrogen and extraction was done following the method of Li et al. (2003). The powder was washed five times in cold phosphate buffer (50 mmol/L, pH 7.2) and extracted twice with 70% ethanol for 1 h at 70 °C. After vacuum drying, cell wall materials were assayed using FTIR. The cell walls of wheat stems were placed on a barium fluoride window supported on the stage of a Nicolet NicPlan IR Microscope accessory of a Nicolet Magna-IR 750 FTIR spectrometer equipped with a liquid nitrogen-cooled mercury cadmium telluride detector. An area of the cell wall (100×100 μm) was selected for the spectral collection. Sixty-four interferograms were collected in transmission mode with 4 cm^−1^ resolutions and co-added to improve the signal-to-noise ratio for each stem internode. Three spectra were collected from internodes of the different stems, and then averaged and baseline-corrected. The triplicate-averaged spectrum was then assayed.

### Statistical analysis

The presented values are the mean ± SD values. The significant differences between the mutant and control plants were indicated by an asterisk based on a student’s *t*-test using R-4.2.1 software (*P < 0.05, **P < 0.01). All the statistical analyses were performed using Microsoft Excel for Windows (Microsoft Corp., Redmond, WA, USA). Correlation matrix were also carried out and to test the significance of correlation coefficients, critical table values were utilized.

## Results

### Comparison of morphological and anatomical features

The morphological differences clearly showed the superiority of PMW-2016-1 over the parent i.e., DPW-621-50 (**Table 1**). The lodging-tolerant mutant PMW-2016-1 was marked by a large outer radius i.e., 2.40 mm of the 2^nd^ internode. The evaluated mutant against the parent DPW-621-50 also showed varied differences in the thickness of the stem wall (SWT) and the ratio of SWT to the outer radius. The thicker stem walls along with a higher SWT/outer radius ratio in the region of the 2^nd^ internode was observed in the mutant. Large vascular bundles were scattered in parenchyma tissues, and small vascular bundles were distributed under the epidermis. The count was comparatively higher in PMW-2016-1 for both types of vascular bundles. Furthermore, the average number of large and small vascular bundles per unit area was also reported more in the mutant, PMW-2016-1 as compared to its parent cultivar, DPW-621-50. There were only minor differences in the culm diameter and internode wall thickness of internodes (**Table 2**).

**Table 1:** Morphological characteristics of parent (DPW-621-50) and lodging tolerant mutant (PMW-2016-1) wheat cultivars.

Trait	DPW-621-50	PMW-2016-1
Number of tillers per plant	10.60	15.53
Plant height (cm)	101.04	106.20
Flag leaf length (cm)	24.14	21.48
Number of spikelets per spike	20.00	23.83
Number of grains per spike	53.77	56.92
Thousand grain weight (g)	38.99	45.10

**Table 2:** Comparison of anatomical features and compositions of 2^nd^ Internode among DPW-621-50 (parent) and PMW-2016-1 (lodging tolerant mutant).

Characteristics	DPW-621-50 (Parent)	PMW-2016-1 (Mutant)
Outer radius (mm)	2.17 ± 0.01	2.4 ± 0.02**
Stem wall thickness (mm)	0.84 ± 0.005	0.98 ± 0.01**
The ratio of the thickness of stem wall to outer radius	0.39 ± 0.005	0.41 ± 0.01
Proportion of sclerenchyma tissue (%)	10.67 ± 3.00	13.57 ± 3.12
Proportion of parenchyma area (%)	71.68 ± 10.20	80.46 ± 11.42
Number of big vascular bundles	23.02 ± 0.37	29.78 ± 0.85**
Number of small vascular bundles	20.49 ± 0.40	22.76 ± 0.26**
Total number of vascular bundles	42.83 ± 0.87	50.58 ± 0.44**
Average number of big vascular bundle per unit area	5.73 ± 0.78	7.04 ± 0.66
Average number of small vascular bundle per unit area	4.4 ± 0.15	5.1 ± 0.22*
Average number of vascular bundles per unit area	9.54 ± 0.46	11.16 ± 0.99
Culm Diameter (mm)	5.54 ± 0.04	6.13 ± 0.41
Internode wall thickness (mm)	0.86 ± 0.10	1.3 ± 0.36
Lignin content	23.63 ± 0.42	28.42 ± 0.25**
Lodging resistance	27.25 ± 0.5	30.21 ± 1.19*
Bending stress (N)	154.87 ± 42.47	264.9 ± 110.3

Data in the table are means ± S.D. levels of significance are: ^*^ p < 0.05, ^**^ p < 0.01.

### Histochemical staining of the stem cell walls

**Figure 1:**
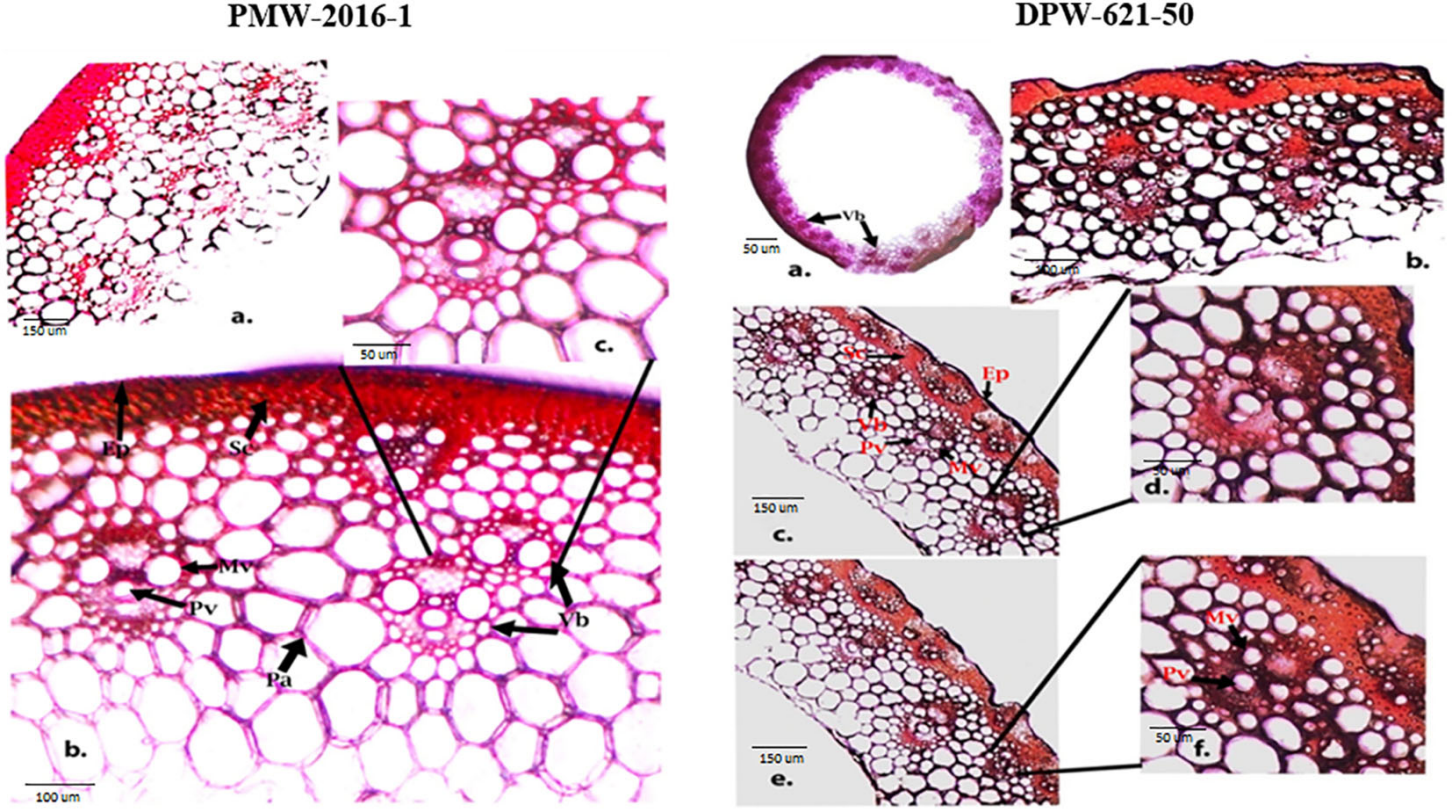
Phloroglucinal-HCl (Wiesner) staining of stem sections of PMW-2016-1 **(A-C)** and DPW-621-50 **(A-F)** showing lignin deposition in the walls of sclerenchyma, Protoxylem and Metaxylem cells. The positions of the Protoxylem (Pv), Metaxylem (Mv), Sclerenchyma (Sc), Parenchyma (Pa), Epidermis (Ep), Vascular bundles (Vb) are indicated.

**Figure 2:**
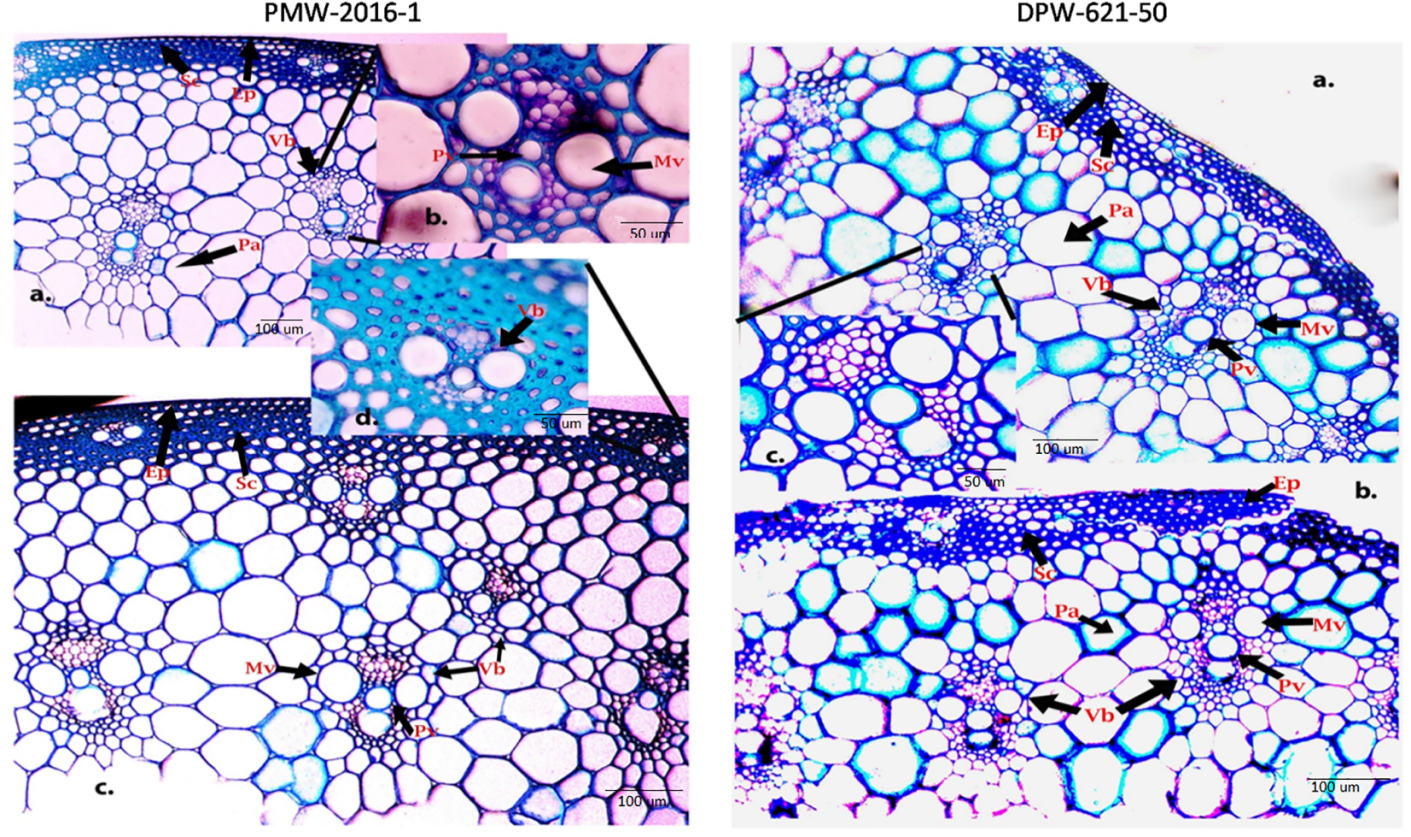
Toluidine blue- O staining of PMW-2016-1 **(A-D)** and DPW-621-50 **(A-C)** stem sections showing lignin deposition in the walls of sclerenchyma, Protoxylem and Metaxylem cells **(A-C)**. The positions of the Protoxylem (Pv), Metaxylem (Mv), Sclerenchyma (Sc), Parenchyma (Pa), Epidermis (Ep), Vascular bundles (Vb) are indicated.

To visualize how lignin concentration varied among the stems of lodging tolerant mutant (PMW-2016-1) and parent (DPW-621-50), transverse sections of PMW-2016-1 and DPW-621-50 were histochemically stained with Wiesner reagents and toluidine blue O. After comparison, we found dark and light stained culm sections from mutant (PMW-2016-1) and parent (DPW-621-50) plants with phloroglucinol–HCl, respectively. Major color differences were found between the mutant (PMW-2016-1) and parent (DPW-621-50) in the mechanical or sclerenchyma tissues, especially the region below the epidermis and vascular bundles. The Wiesner reaction was able to distinguish the differences in lignin compounds of different stems of wheat. Red coloration was seen in the sclerenchyma tissues and vascular bundles. The parenchyma tissues appeared pink, slowly from inside to outside. A noticeable dark red staining appeared in PMW-2016-1, but weak staining was found in DPW-621-50 (**Figures 1**, **2**).

### Lignin content in stem cell wall

**Figure 3:**
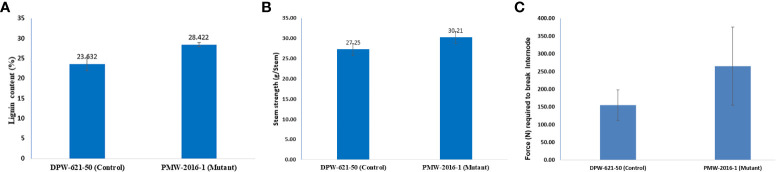
Results of **(A)** Lignin content **(B)** stem strength **(C)** bending stress, of 2^nd^ internodes of lodging tolerant mutant (PMW-2016-1) and parent (DPW-621-50) wheat cultivars.

The Klason lignin content in the cell wall of stems of putative lodging-tolerant mutant (PMW-2016-1) and parent (DPW-621-50) was found to be 28.42% and 23.63%, respectively. The lignin content in the PMW-2016-1 stem was significantly higher than that in DPW-621-50 (**Figure 3A** and **Table 2**).

### Estimation of mechanical stem strength using a prostate tester and UTM

Mechanical strength was estimated by analysing the stem bending of mutant (PMW-2016-1) and parent (DPW-621-50) using a prostate tester. The ranking of the strength was done based on stem resistance to pushing which was performed by the prostate tester on the bottom part of the stem. The average stem strength for PMW-2016-1 was recorded as 30.21 g/stem, found to be significantly higher than that of DPW-621-50 (27.25 g/stem) (**Figure 3B**). By comparing the internode thickness and force (N) required to break the internodes of the mutant (PMW-2016-1) and the parent (DPW-621-50), we observed that the newly identified mutant showed more mechanical strength and have thicker internode walls (**Table 2**). Universal tensile machine results reported that the force required to break the 2^nd^ internode of the mutant PMW-2016-1 i.e., 264.9 N was significantly higher than that of the parent DPW- 621-50 i.e., 154.87 N (**Figure 3C**).

### Fourier transform infrared spectroscopy analysis

The FTIR spectra at 800 to 1800 cm^-1 are^ the most interesting region that identifies different chemical groups. The different spectral peaks provide different functional groups i.e., carbonyl C=O at 1747, amide I C=O at 1668, aromatic rings of lignin (may be ferulic acid) at 1612, aromatic skeletal vibrations at 1516, lignin at 1462 & 1425, CH band at 1381, lignin at 1320, amide III in protein at 1246, CHO at 1163, 1059 & 899 cm^-1^. The different functional groups were assigned according to earlier studies (Sene et al., 1994; McCann et al., 1994; Agarwal et al., 1995; Stewart et al., 1997; Zhong et al., 2000).

**Figure 4:**
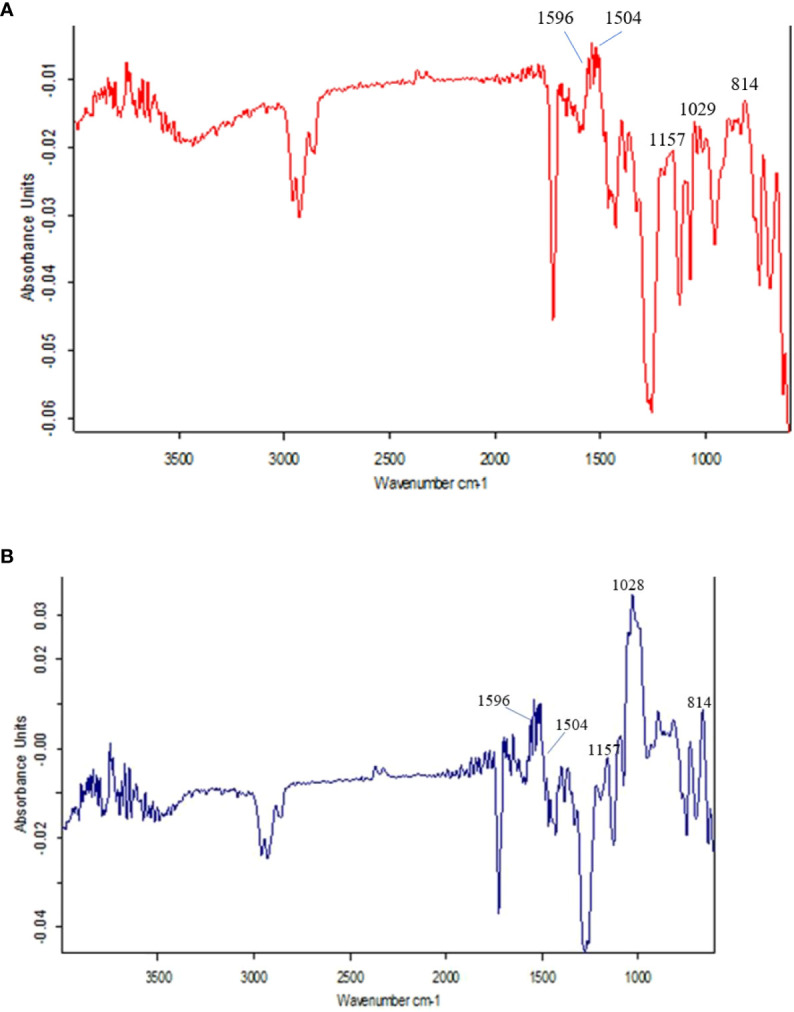
Results of FTIR spectra of the components in cell wall of **(A)** lodging tolerant mutant PMW-2016-1 and **(B)** parent DPW 621-50 wheat cultivars.

Here, in the present investigation, we compared and assayed the components of cell walls of PMW-2016-1 and its parent DPW-621-50 stem using FTIR. In the present investigation, the variations in the intensity of some bands in the spectra reflect compositional differences between the cell walls of mutant PMW-2016-1 and its parent DPW-621-50. **Figures 4A, B** represent the FTIR spectra collected from the fibers in mechanical tissue of the newly identified lodging tolerant mutant PMW-2016-1 and its parent DPW-621-50. In several previous studies, it has been reported that the comparative degree of lignin present in the cell walls can be crudely assessed by the ratio of the peaks at 1505―1596 cm^−1^ for the two absorbances, characteristics of lignin or lignin-like structures (McCann et al., 1994; Zhong et al., 2000). Although the FTIR spectra of the cell walls of PMW-2016-1 and its parent DPW-621-50 stems were essentially similar, but slight differences can be noted. For example, the relative absorbances in the 1200―900 cm^−1^ region, and the principal absorbance regions for polysaccharide absorbance are different among the PMW-2016-1 and its parent DPW-621-50 stem cell walls. Differences in the FTIR spectra of lignin absorbance clearly showed that the peak of the lignin at the 1540- 1567 cm^−1^ region was higher in PMW-2016-1 than those in the parent DPW-621-50 stem cell walls. The ratio of 1504:1596 cm^-1^ was found more in PMW-2016-1 as compared to control DPW-621-50. In DPW-621-50, the ratio was reduced to 62% of PMW-2016-1 (**Table 3**).

**Table 3:** Comparison of lignin absorbance from the FTIR spectra of the two different genotypes of wheat stem cell walls illustrating the maximum lignin content in PMW-2016-1.

Genotype	Absorbance at 1504 cm^-1^	Absorbance at 1596 cm^-1^	1504/1596 Absorbance ratio
DPW-621-50	-0.00611	-0.0077	0.7935
PMW-2016-1	-0.0153	-0.01192	1.2835

### Correlation of stem characteristics with lodging resistance

The lodging resistance of PMW-2016-1 was 1.11-fold that of DPW-621-50 (**Table 2**). The highest positive correlation (0.9648) was found between lodging resistance and culm diameter, and the lowest (0.0692) was found between lodging resistance and parenchymatous tissue proportion. Lodging resistance was positively correlated with OR (r =0.920, p < 0.01), SWT (r = 0.917, p < 0.01), NBV (r =0.953, p < 0.01), NSV (r = 0.903, p < 0.05), TV (r = 0.888, p < 0.05), ASVA (r = 0.868, p < 0.05), CD (r = 0.965, p < 0.01), IWT (r = 0.880, p < 0.05), BS (r = 0.910, p < 0.05), and Lignin (r = 0.929, p < 0.01) (**Table 4**). In addition, highly significant positive correlations (p < 0.01) were found between OR and SWT (r = 0.998), OR and NBV (r = 0.992), OR and NSV (r = 0.978), OR and TV (r = 0.996), OR and ASVA (r = 0.926), OR and Lignin (r = 0.987), SWT and NBV (r = 0.992), SWT and NSV (r = 0.986), SWT and TV (r = 0.996), SWT and ASVA (r = 0.935), SWT and Lignin (r = 0.989), NBV and NSV (r = 0.971), NBV and TV (r = 0.978), NBV and ASVA (r = 0.921), NBV and Lignin (0.996), NSV and TV (r = 0.982), NSV and ASVA (r = 0.956), NSV and Lignin (r = 0.962), TV and ASVA (r = 0.931), TV and Lignin (r = 0.974), ABVA and IWT (r = 0.919), CD and IWT (r = 0.963); and significant positive correlations (p < 0.05) were found between OR and CD (r = 0.833), SWT and CD (r = 0.825), NBV and CD (r = 0.862), ST and ABVA (r = 0.816), ST and IWT (r = 0.847), NSV and ABVA (r = 0.829), NSV and CD (r = 0.830), TV and ABVA (r = 0.828), ABVA and CD (r = 0.815), ASVA and Lignin (r = 0.907), CD and Lignin (r = 0.817), and, IWT and BS (r = 0.882). Whereas no significant correlations were found between lodging resistance and RSO, ST, PT, ABVA, and ATVA. Negative correlations were also found between PT and CD, IWT, BS, ST, and ATVA (**Table 4**).

**Table 4:** Correlation coefficients for lodging resistance and stem characteristics of PMW-2016-1 and DPW-621-50 used as a control.

	OR	SWT	RSO	ST	PT	NBV	NSV	TV	ABVA	ASVA	ATVA	CD	IWT	BS	LIGNIN
SWT	0.9984**														
RSO	0.7573	0.7696													
ST	0.5640	0.5780	0.3925												
PT	0.3739	0.4114	0.6888	0.1644											
NBV	0.9921**	0.9924**	0.7155	0.5641	0.3255										
NSV	0.9779**	0.9860**	0.7785	0.6874	0.4655	0.9714**									
TV	0.9959**	0.9956**	0.7897	0.5796	0.4337	0.9784**	0.9824**								
ABVA	0.8056	0.7955	0.6010	0.8161*	0.1937	0.7613	0.8288*	0.8275*							
ASVA	0.9261**	0.9347**	0.5965	0.6985	0.4314	0.9212**	0.9559**	0.9309**	0.8088						
ATVA	0.7144	0.7278	0.6969	-0.0385	0.6465	0.7160	0.6682	0.7114	0.2111	0.6008					
CD	0.8332*	0.8247*	0.4224	0.7589	-0.0890	0.8623*	0.8300*	0.8030	0.8154*	0.8096	0.2823				
IWT	0.7834	0.7720	0.4258	0.8467*	-0.0747	0.7857	0.7979	0.7719	0.9195**	0.7858	0.1329	0.9630**			
BS	0.6827	0.6739	0.2175	0.6712	-0.2900	0.7417	0.6735	0.6321	0.6352	0.6632	0.1631	0.9594**	0.8815*		
LIGNIN	0.9873**	0.9892**	0.7328	0.5015	0.3721	0.9957**	0.9619**	0.9736**	0.7101	0.9074*	0.7765	0.8166*	0.7258	0.6948	
LR	0.9203**	0.9166*	0.5375	0.6471	0.0692	0.9527**	0.9028*	0.8884*	0.7515	0.8676*	0.5194	0.9648**	0.8800*	0.9103*	0.9294**

OR, outer radius; SWT, Stem wall thickness; RSO, The ratio of the thickness of stem wall to outer radius; ST, Proportion of sclerenchyma tissue (%); PT, Proportion of parenchyma area (%); NBV, Number of big vascular bundles; NSV, Number of small vascular bundles; TV, Total number of vascular bundles; ABVA, Average number of big vascular bundle per unit area; ASVA, Average number of small vascular bundle per unit area; ATVA, Average number of vascular bundles per unit area; CD, Culm Diameter; IWT, Internode wall thickness; BS, Bending stress; LR, Lodging resistance.

^*^ Significant at p < 0.05, ^**^ Significant at p < 0.01.

## Discussion

Lodging forces often distort the physical skeleton of the wheat plant, which further results in the failure of the vascular system to effectively mobilize the stem reserves to sink, which affects grain size, grain weight, etc. thereby resulting in yield loss (Shah et al., 2017). Khanna (1991) observed a strong relationship between the number of vascular bundles and lodging resistance. The author demonstrated that the larger number of vascular bundles works as a bypass for the broken routes of the xylem and phloem tissues, which results in better recovery and grain filling. Therefore, a larger diameter and a solid stem could be used as traits of choice for an increasing number of vascular bundles. Several studies have demonstrated that stem strength is associated with its diameter and wall thickness (Kokubo et al., 1989). Stem length, number of internodes, and density of stem also affect the stem strength (Zhong et al., 1997; Kaack et al., 2003; Kashiwagi and Ishimaru, 2004). It is also observed that the stem wall thickness in wheat is strongly related to lodging resistance (Kokubo et al., 1989). In contrast, it has also been reported that stem strength decreases with an increase in stem diameter (Norden and Frey, 1959; Dunn and Briggs, 1989). Some researchers have suggested that the larger diameter and wall thickness of basal internodes of the stem may be good criteria for the development of lodging-resistant cultivars (Ross et al., 1998; Xiao et al., 2002; Tripathi et al., 2003). In the present study, we found that the stems of a newly developed mutant (PMW-2016-1) possess solid stems, larger stem diameter, higher stem wall thickness, and a significantly higher number of large vascular bundles per unit area than its parent (DPW-621-50). We also found that the different anatomical characteristics such as stem diameter, wall thickness, and the number of vascular bundles have a significant positive correlation with the stem strength/lodging resistance.

The mechanical tissues i.e., sclerenchyma tissues and vascular bundles, provide mechanical strength to the plant (Fahn, 1982) and these tissues were the basis of brittle and non-brittle stems in rice (Li et al., 2003). Ansari et al. (2013) reported that in a brittle culm mutant (brc5), sclerenchyma cells are hollow and thinner with little deposition of cell wall materials. This study also suggested that epidermal cells provide the plant’s stem strength. Mutant brc5 epidermal cells are thinner due to the lack of cell wall depositions. In contrast to wild-type (WT) culm, mutant brc5 culm showed uneven shapes of some parenchyma cells. On comparing the culms of PMW-2016-1 and DPW-621-50, we found that the stems of PMW-2016-1 have a larger number of vascular bundles per unit area and a higher fraction of sclerenchyma tissues. Our study provided a positive correlation between the proportion of sclerenchyma tissue and the lodging resistance, but the relationship was not so significant. The positive correlations of mechanical tissues indicate their role in providing lodging resistance in wheat.

Lignin is a key structural material of the secondary cell wall that is akin to plant growth and provides mechanical support to plants. The contents of lignin and cellulose were found to be higher in the sclerenchyma tissues and vascular bundles (Kong et al., 2013). A positive correlation was found in wheat between lignin content and stem strength. Less lignin accumulation results in the higher vulnerability of plants to lodging. Cultivars that accumulate more lignin could be a viable alternative to impart heritable mechanical strength (Berry et al., 2003; Peng et al., 2014). Therefore, the assay for the chemical composition of wheat stems offers a better phenotyping tool for improving lodging resistance, and this strategy has been used successfully in many studies. On comparing the culms of PMW-2016-1 and DPW-621-50, we found that the stems of PMW-2016-1 have a higher lignin content. Also, a significantly higher positive correlation was found between lignin content and lodging resistance (r = 0.9294). Therefore, the comparison suggested that lodging resistance/stem strength in mutant PMW-2016-1 also occurred due to increased lignin accumulation in the stem cell walls.

Fourier transform infrared spectroscopy **(**FTIR) is a highly reproducible and reliable means to investigate cell wall compositional differences (Séné et al., 1994; McCann et al., 1994; Agarwal et al., 1995; Stewart et al., 1997; Zhong et al., 2000) and further highlight the subtle differences in components of stem cell walls (Kashiwagi and Ishimaru, 2004) or mechanical strength changes in stems (Wilson et al., 2000). In this study, the observed absorbance peaks at different wavenumbers are associated with concentrations of different molecules of cell wall materials such as cellulose, lignin, pectin, and proteins. The higher ratio of 1504:1596 cm^-1^ in PMW-2016-1 provided a quantitative measure of more condensed and cross-linked lignin as compared to control DPW-621-50 (**Table 3**). Differences in the FTIR spectra of lignin absorbance clearly showed that the peak of the lignin at the 1540- 1567 cm^−1^ region was higher in PMW-2016-1 than those in the parent DPW-621-50 stem cell walls, suggesting a relatively higher lignin content in the former. Nevertheless, this finding agreed well with those gained by mechanical, anatomical observations, and Histochemical staining.

We observed that the newly identified mutant showed more mechanical strength and thicker internode walls (**Table 2**). The correlation analysis also provided significant positive relations of lodging resistance to the culm diameter, internode thickness, and the bending stress required to break the internodes. The significant increase in the breaking force of the mutant (PMW-2016-1) suggested that mutation in PMW-2016-1 strongly affects the mechanical strength of culmsbut these results are difficult to explain. The amount of lignin present in the organic matter could be one of the possible reasons (Kriauciuniene et al., 2012). From the literature survey, it was stated that the content of certain materials such as lignin, hemicellulose, and cellulose vary in different types of straw (Grabber, 2005; Kriauciuniene et al., 2012; Sarauskis et al., 2013), which could also impact the resistance to stem breakage. The environmental factors alter the physical, biological and mechanical characteristics of the stem present on and above the soil surface. Lodging might be reduced by a thicker tiller that allows a significant flow of water and nutrients in the plant. It was reported that the diameter and specific density of stems are positively correlated to lodging and stem strength (Zuber et al., 1999).

Along with the thickness of the stem cell wall, the thickness of the pith parenchyma also determines the mechanical resistance against stem bending (Ennos, 1993; Spatz et al., 1998). The lignin content and its minuteness, carbon to nitrogen ratio determine the intensity of cereal straw breakage (Kriauciuniene et al., 2012). Solid-stem wheat cultivars tend to have higher lodging tolerance than hollow-stem wheat cultivars (Kong et al., 2013). It might be possible that the mutant PMW-2016-1 might have some modification in the biosynthesis of the secondary cell wall and have higher pith thickness, resulting in thick stem walls. The preliminary study on mutant PMW-2016-1 suggests that this plant can be used as a variety or as a donor parent for the development of lodging tolerance wheat with higher biomass.

## Conclusion

We investigated the structural characteristics and cell wall composition of the stems of a putative lodging-tolerant mutant PMW-2016-1 and parent DPW-621-50. The stem mechanical tissues present in the outer ring, and the pith parenchyma tissues present in the inner ring of the stem increase the lodging resistance. Considering our findings obtained from histochemical staining, it is reasonable to propose that the ratio of the outer radius of the stem and thickness of the stem wall, a large proportion of sclerenchyma tissue, a large number of vascular bundles, a large number of average vascular bundles per unit area, and lignification are highly related to lodging resistance. Our study suggests that the prior mechanical and anatomical characteristics, as well as high lignin content in the culm of the newly developed mutant PMW-2016-1, may enhance the role of support and lodging resistance. Therefore, it is suggested that the newly identified mutant PMW-2016-1 can be used as a variety or as a donor parent for the development of lodging tolerance in wheat cultivars.

## Data availability statement

The original contributions presented in the study are included in the article/supplementary material. Further inquiries can be directed to the corresponding authors.

## Author contributions

SK and UK conceived the idea. DB and NK carried out the experiments. YS, PB, VS, ND, NP, RRM and RM analysed the data. SK, UK, YS, ID, OPD and RRM drafted and edited the manuscript. All authors contributed to the article and approved the submitted version.

## Funding

This study was funded by the Department of Biotechnology (DBT) and the Government of India for financial support (BT/NABI-Flagship/2018). The author SK has received the grant.

## Acknowledgments

Thanks are due to the Department of Molecular Biology and Genetic Engineering, College of Basic Sciences and Humanities and Norman E Borlaug Crop Research Centre, G. B. Pant University of Agriculture and Technology, Pantnagar for conducting field experiments.

## Conflict of interest

The authors declare that the research was conducted in the absence of any commercial or financial relationships that could be construed as a potential conflict of interest.

## Publisher’s note

All claims expressed in this article are solely those of the authors and do not necessarily represent those of their affiliated organizations, or those of the publisher, the editors and the reviewers. Any product that may be evaluated in this article, or claim that may be made by its manufacturer, is not guaranteed or endorsed by the publisher.
